# The genome sequence of an ichneumonid wasp, *Netelia fuscicornis* (Holmgren, 1860)

**DOI:** 10.12688/wellcomeopenres.22578.1

**Published:** 2024-07-10

**Authors:** Benjamin W. Price, Gavin R. Broad, Natural History Museum Genome Acquisition Lab, Darwin Tree of Life Barcoding collective, Wellcome Sanger Institute Tree of Life programme, Wellcome Sanger Institute Scientific Operations: DNA Pipelines collective, Tree of Life Core Informatics collective, Darwin Tree of Life Consortium

**Affiliations:** 1 Natural History Museum, London, England, UK

**Keywords:** Netelia fuscicornis, ichneumonid wasp, genome sequence, chromosomal, Hymenoptera

## Abstract

We present a genome assembly from an individual female *Netelia fuscicornis* (an ichneumonid wasp; Arthropoda; Insecta; Hymenoptera; Ichneumonidae). The genome sequence is 324.7 megabases in span. Most of the assembly is scaffolded into six chromosomal pseudomolecules. The mitochondrial genome has also been assembled and is 17.7 kilobases in length.

## Species taxonomy

Eukaryota; Opisthokonta; Metazoa; Eumetazoa; Bilateria; Protostomia; Ecdysozoa; Panarthropoda; Arthropoda; Mandibulata; Pancrustacea; Hexapoda; Insecta; Dicondylia; Pterygota; Neoptera; Endopterygota; Hymenoptera; Apocrita; Ichneumonoidea; Ichneumonidae; Tryphoninae; Phytodietini; *Netelia*; *Netelia fuscicornis* (Holmgren, 1860)(NCBI:txid2803881).

## Background

*Netelia fuscicornis* is a fairly common and widespread ichneumonid wasp, one of about 45 European species of *Netelia*, the vast majority of which are nocturnal and pale reddish. Identification of Netelia species is often difficult but *N. fuscicornis* can be identified by a combination of dark antennae in males (which is the meaning of the species name), strong scutellar carinae, details of the fore wing venation, head shape and the internal structures of the male genitalia ( Delrio, 1974). Males of *Netelia* are unusual in Ichneumonidae as the genitalia have large membranous pads on the internal surface of the gonosquama, articulated via a strip of cuticle called the ‘brace’, and the shapes of these structures are important in identification ( Dal Pos *et al.*, 2023; Delrio, 1974; Townes, 1939). Identification of *N. fuscicornis* is complicated by variation in appearance through the year. There are apparently two or more generations per year, with earlier flying specimens (such as that sequenced here) having wider temples (the top of the head) than the narrower-headed specimens flying later in the year, typically in September. Whether these are actually conspecific has not been tested, although there is no separation in DNA barcode data (G. Broad, unpublished). A morphologically similar species, *Netelia dilatata*, is active in May on sand dunes and has often been confused with *N. fuscicornis*.

All species of *Netelia* which have been reared are koinobiont ectoparasitoids of Lepidoptera larvae ( Broad *et al.*, 2018). The female stings and temporarily paralyses the caterpillar, anchoring an egg in the host cuticle. The *Netelia* larva completes most of its development, sucking haemolymph from the host, once the caterpillar has formed a pupation retreat (see ( Shaw, 2001; Shevyrev, 1912). *Netelia fuscicornis* is gregarious and in Britain has been reared in small broods from larvae of noctuid moths which feed at ground level and pupate in the soil, such as *Mythmina* (Wainscot moths) (G. Broad, unpublished). As a consequence, *N. fuscicornis* is most frequently found in open areas. Delrio (1974) notes an extensive range across Europe and as far East as China, although Konishi ( Konishi, 2005) found that records from Japan were unreliable.

This genome adds to a small but growing number of *Netelia* genomes and will aid in comparative analyses of traits associated with the evolution of host range within this morphologically conservative but ecologically varied group of wasps.

## Genome sequence report

**Figure 1.**
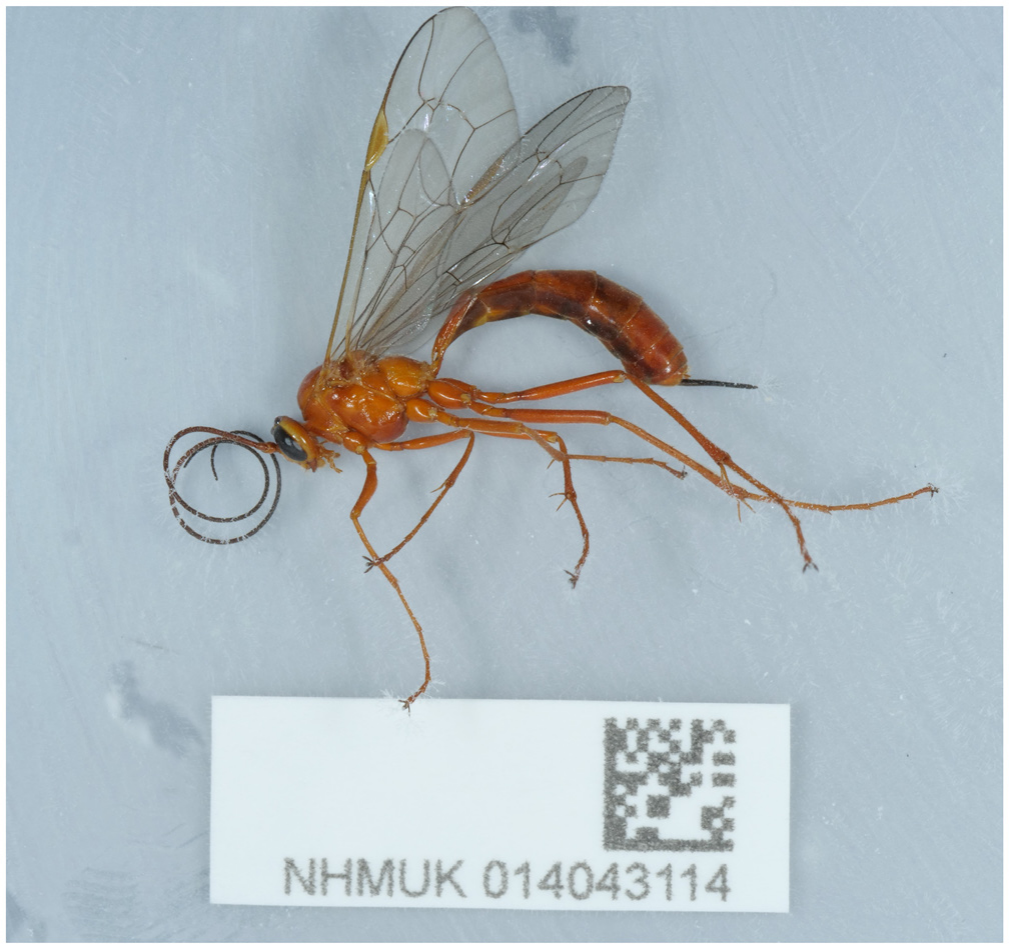
Photograph of the *Netelia fuscicornis* (iyNetDila1) specimen used for genome sequencing.

The genome was sequenced from one female *Netelia fuscicornis* specimen ( Figure 1) collected from Pulborough, UK (latitude 50.96, longitude –0.51). A total of 63-fold coverage in Pacific Biosciences single-molecule HiFi long reads was generated. Primary assembly contigs were scaffolded with chromosome conformation Hi-C data. Manual assembly curation corrected 76 missing or mis-joins and removed four haplotypic duplications, reducing the scaffold number by 7.48% and increasing the scaffold N50 by 120.57%.

**Figure 2.**
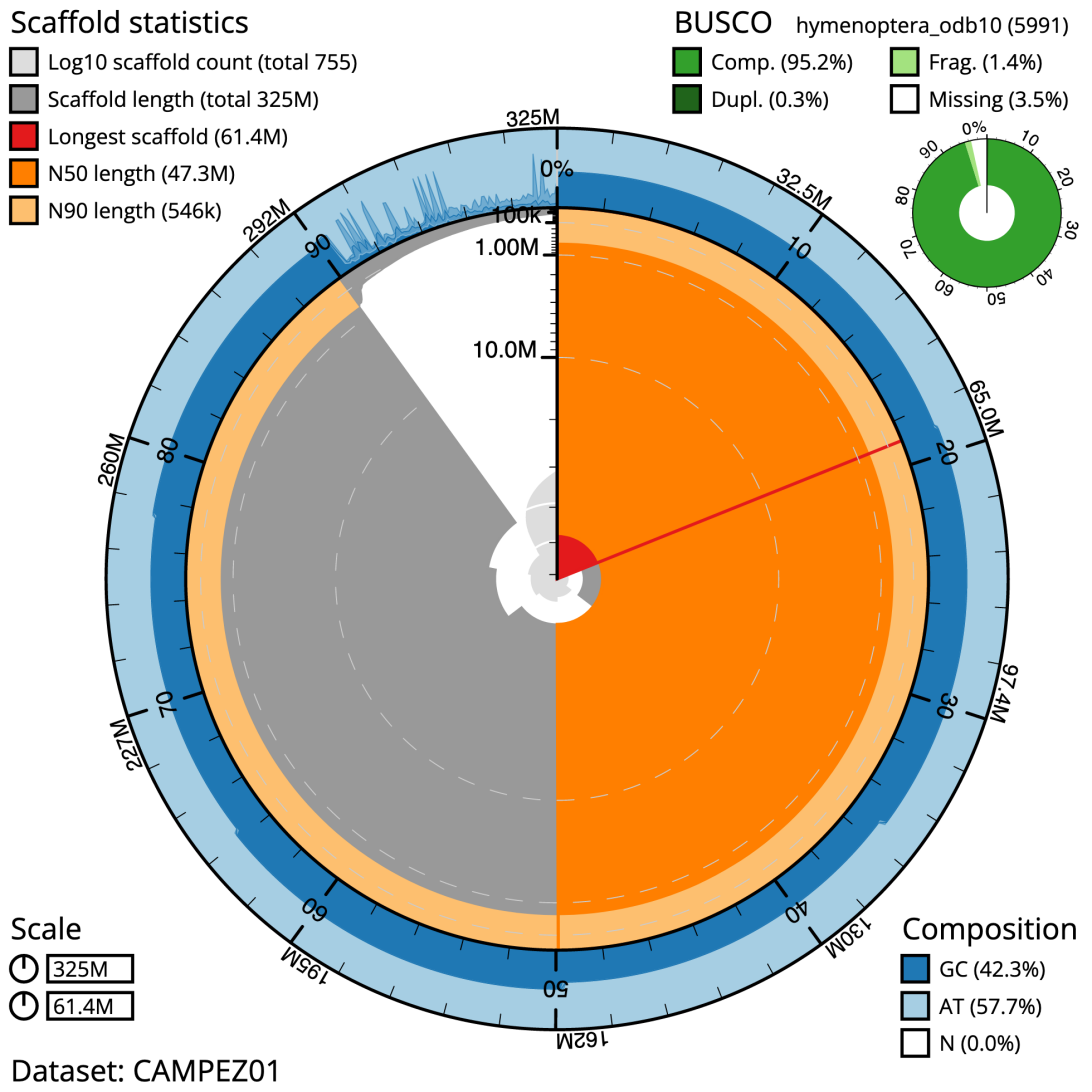
Genome assembly of *Netelia fuscicornis*, iyNetDila1.1: metrics. The BlobToolKit Snailplot shows N50 metrics and BUSCO gene completeness. The main plot is divided into 1,000 size-ordered bins around the circumference with each bin representing 0.1% of the 324,758,365 bp assembly. The distribution of scaffold lengths is shown in dark grey with the plot radius scaled to the longest sequence present in the assembly (61,433,639 bp, shown in red). Orange and pale-orange arcs show the N50 and N90 scaffold lengths (47,279,114 and 546,000 bp), respectively. The pale grey spiral shows the cumulative scaffold count on a log scale with white scale lines showing successive orders of magnitude. The blue and pale-blue area around the outside of the plot shows the distribution of GC, AT and N percentages in the same bins as the inner plot. A summary of complete, fragmented, duplicated and missing BUSCO genes in the hymenoptera_odb10 set is shown in the top right. An interactive version of this figure is available at https://blobtoolkit.genomehubs.org/view/iyNetDila1.1/dataset/CAMPEZ01/snail.

**Figure 3.**
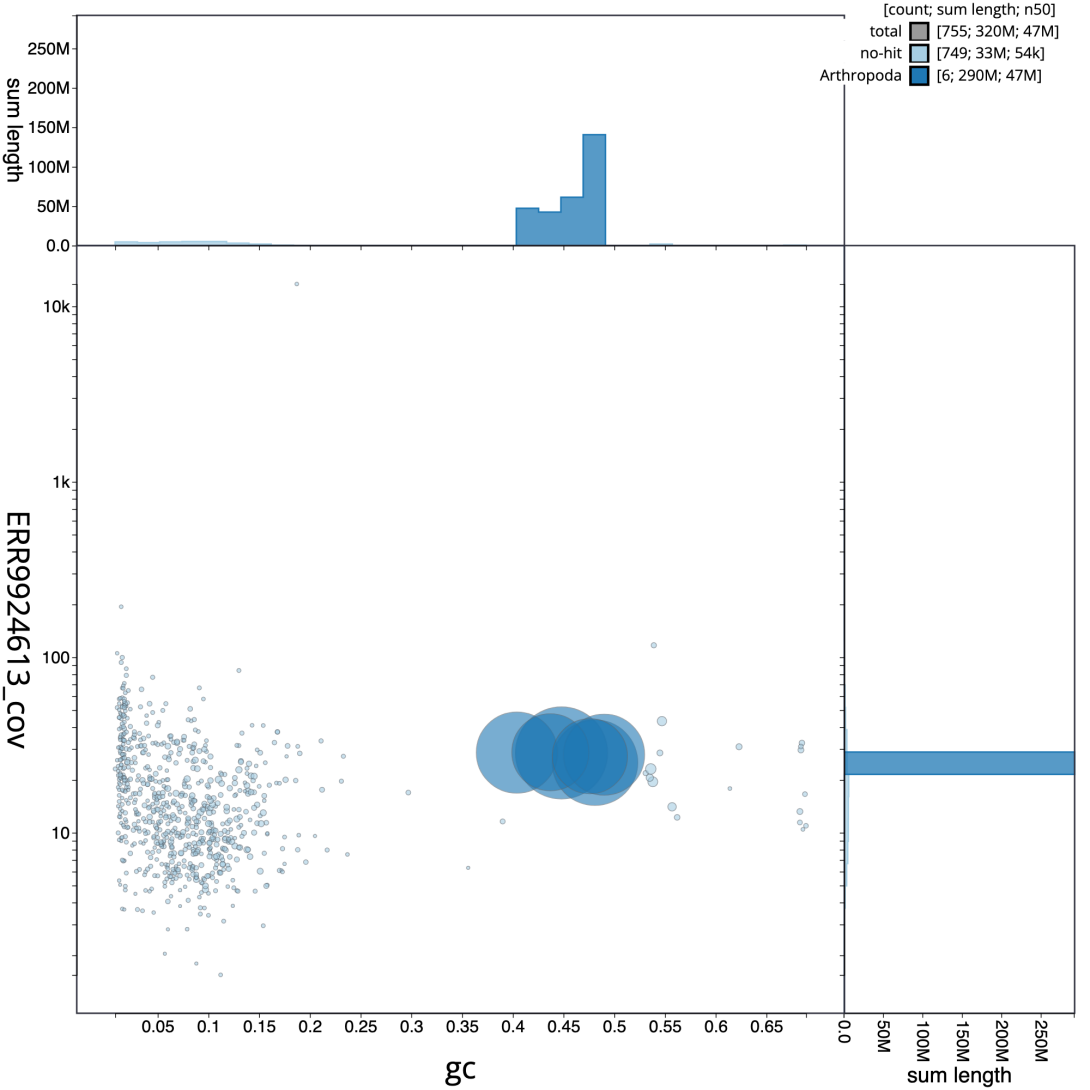
Genome assembly of *Netelia fuscicornis*, iyNetDila1.1: GC coverage. BlobToolKit GC-coverage plot. Scaffolds are coloured by phylum. Circles are sized in proportion to scaffold length. Histograms show the distribution of scaffold length sum along each axis. An interactive version of this figure is available at https://blobtoolkit.genomehubs.org/view/iyNetDila1.1/dataset/CAMPEZ01/blob.

**Figure 4.**
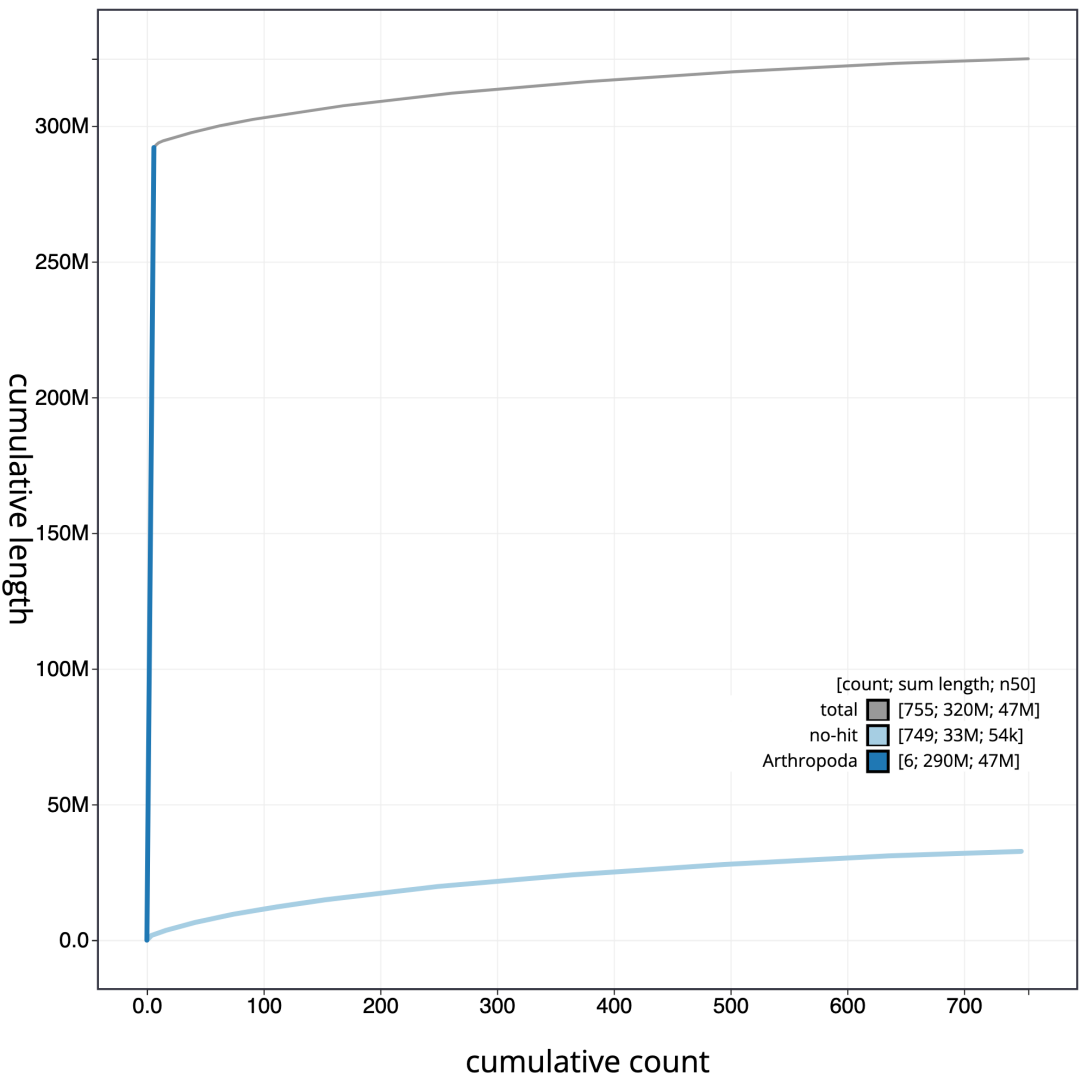
Genome assembly of *Netelia fuscicornis*, iyNetDila1.1: cumulative sequence. BlobToolKit cumulative sequence plot. The grey line shows cumulative length for all scaffolds. Coloured lines show cumulative lengths of scaffolds assigned to each phylum using the buscogenes taxrule. An interactive version of this figure is available at https://blobtoolkit.genomehubs.org/view/iyNetDila1.1/dataset/CAMPEZ01/cumulative.

**Figure 5.**
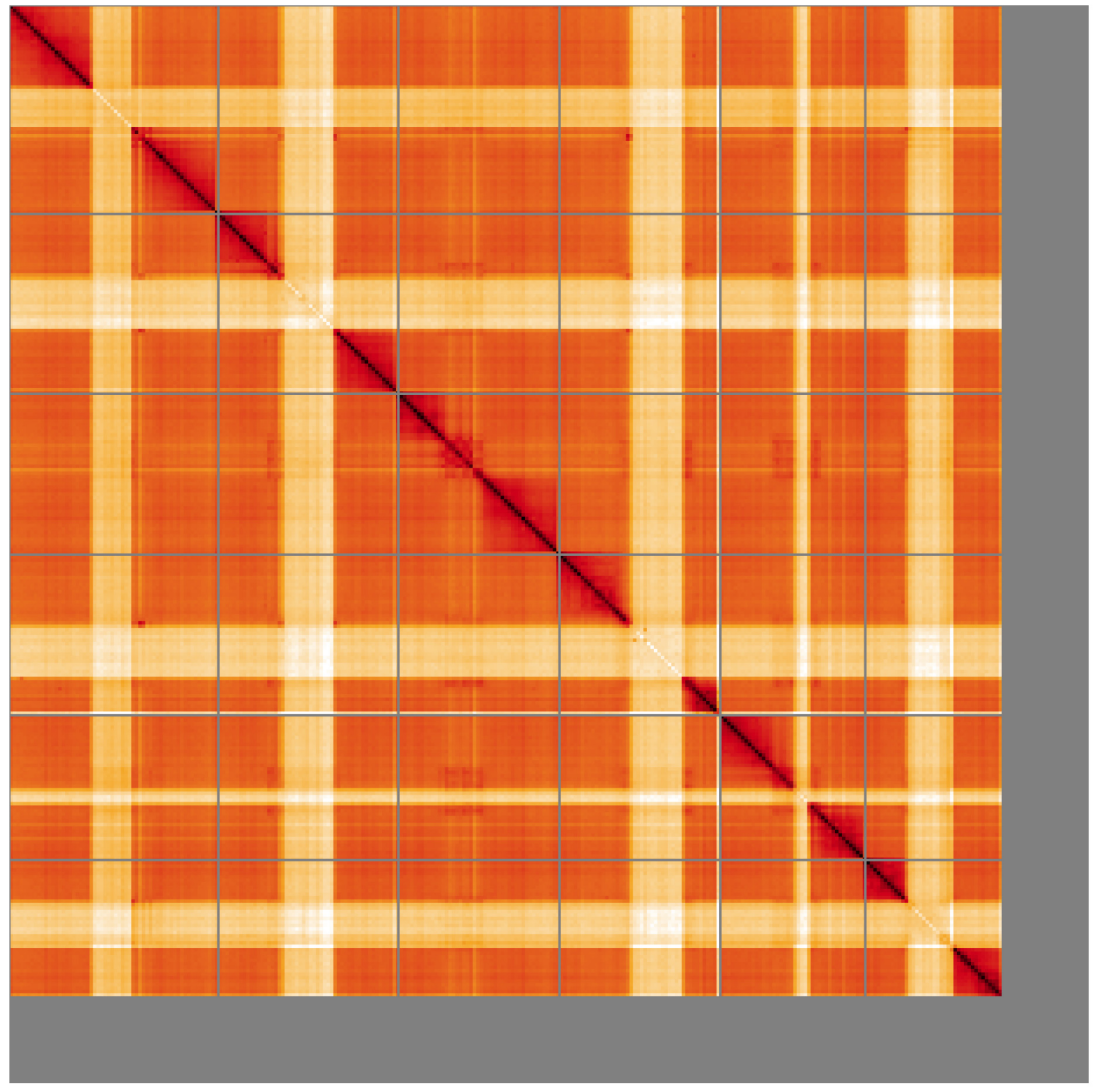
Genome assembly of *Netelia fuscicornis*, iyNetDila1.1: Hi-C contact map. Hi-C contact map of the iyNetDila1.1 assembly, visualised using HiGlass. Chromosomes are shown in order of size from left to right and top to bottom. An interactive version of this figure may be viewed at https://genome-note-higlass.tol.sanger.ac.uk/l/?d=dGp1BP55SvWWU3KRW92KAg.

The final assembly has a total length of 324.7 Mb in 754 sequence scaffolds with a scaffold N50 of 47.3 Mb ( Table 1). The snail plot in Figure 2 provides a summary of the assembly statistics, while the distribution of assembly scaffolds on GC proportion and coverage is shown in Figure 3. The cumulative assembly plot in Figure 4 shows curves for subsets of scaffolds assigned to different phyla. Most (89.92%) of the assembly sequence was assigned to 6 chromosomal-level scaffolds. Chromosome-scale scaffolds confirmed by the Hi-C data are named in order of size ( Figure 5; Table 2). There is a region of undetermined order and orientation on Chromosome 4 from 22,000 kbp to 37,000 kbp. While not fully phased, the assembly deposited is of one haplotype. Contigs corresponding to the second haplotype have also been deposited. The mitochondrial genome was also assembled and can be found as a contig within the multifasta file of the genome submission.

**Table 1.** Genome data for *Netelia fuscicornis*, iyNetDila1.1.

Project accession data		
Assembly identifier	iyNetDila1.1	
Species	*Netelia fuscicornis*	
Specimen	iyNetDila1	
NCBI taxonomy ID	2803881	
BioProject	PRJEB54053	
BioSample ID	Genome sequencing: SAMEA10241745 Hi-C scaffolding: SAMEA10241751 RNA sequencing: SAMEA10241732	
Isolate information	iyNetDila1 female, thorax (genome sequence), head (Hi-C sequencing), abdomen (RNA sequencing)	
Assembly metrics *		*Benchmark*
Consensus quality (QV)	57.9	*≥ 50*
*k*-mer completeness	99.99%	*≥ 95%*
BUSCO **	C:95.2%[S:94.9%,D:0.3%], F:1.4%,M:3.5%,n:5,991	*C ≥ 95%*
Percentage of assembly mapped to chromosomes	89.92%	*≥ 95%*
Sex chromosomes	N/A	*localised * *homologous * *pairs*
Organelles	Mitochondrial genome assembled	*complete * *single alleles*
Raw data accessions		
PacificBiosciences SEQUEL II	ERR9924613	
Hi-C Illumina	ERR9930688	
PolyA RNA-Seq Illumina	ERR10890691	
Genome assembly		
Assembly accession	GCA_946811545.1	
*Accession of alternate haplotype*	GCA_946815625.1	
Span (Mb)	324.8	
Number of contigs	920	
Contig N50 length (Mb)	4.0	
Number of scaffolds	755	
Scaffold N50 length (Mb)	47.3	
Longest scaffold (Mb)	61.4	

* Assembly metric benchmarks are adapted from column VGP-2020 of “Table 1: Proposed standards and metrics for defining genome assembly quality” from ( Rhie *et al.*, 2021). ** BUSCO scores based on the hymenoptera_odb10 BUSCO set using v5.3.2. C = complete [S = single copy, D = duplicated], F = fragmented, M = missing, n = number of orthologues in comparison. A full set of BUSCO scores is available at https://blobtoolkit.genomehubs.org/view/iyNetDila1.1/dataset/CAMPEZ01/busco.

**Table 2.** Chromosomal pseudomolecules in the genome assembly of *Netelia fuscicornis*, iyNetDila1.

INSDC accession	Chromosomes	Size (Mb)	GC%
OX328011.1	1	61.43	44.8
OX328012.1	2	52.92	48.1
OX328013.1	3	47.36	40.4
OX328014.1	4	47.28	49
OX328015.1	5	42.61	43.7
OX328016.1	6	40.45	47.6
OX328017.1	MT	0.02	18.8

The estimated Quality Value (QV) of the final assembly is 57.9 with *k*-mer completeness of 99.99%, and the assembly has a BUSCO v5.3.2 completeness of 95.2% (single = 94.9%, duplicated = 0.3%), using the nan reference set ( *n* = 5,991).

Metadata for specimens, barcode results, spectra estimates, sequencing runs, contaminants and pre-curation assembly statistics are given at https://links.tol.sanger.ac.uk/species/2803881.

## Methods

### Sample acquisition and nucleic acid extraction

A female *Netelia fuscicornis* specimen (specimen ID NHMUK014043114, ToLID iyNetDila1) was collected from Pulborough, UK (latitude 50.96, longitude –0.51) on 3 June 2021, using a light trap. The specimen was collected by Benjamin Price and identified by Gavin Broad (both Natural History Museum, London). The specimen was preserved at –80°C.

The workflow for high molecular weight (HMW) DNA extraction at the Wellcome Sanger Institute (WSI) Tree of Life Core Laboratory includes a sequence of core procedures: sample preparation; sample homogenisation, DNA extraction, fragmentation, and clean-up. In sample preparation, the iyNetDila1 sample was weighed and dissected on dry ice ( Jay *et al.*, 2023). For sample homogenisation, thorax tissue was cryogenically disrupted using the Covaris cryoPREP ^®^ Automated Dry Pulverizer ( Narváez-Gómez *et al.*, 2023).

HMW DNA was extracted using the Automated MagAttract v1 protocol ( Sheerin *et al.*, 2023). DNA was sheared into an average fragment size of 12–20 kb in a Megaruptor 3 system with speed setting 30 ( Todorovic *et al.*, 2023). Sheared DNA was purified by solid-phase reversible immobilisation ( Strickland *et al.*, 2023): in brief, the method employs a 1.8X ratio of AMPure PB beads to sample to eliminate shorter fragments and concentrate the DNA. The concentration of the sheared and purified DNA was assessed using a Nanodrop spectrophotometer and Qubit Fluorometer and Qubit dsDNA High Sensitivity Assay kit. Fragment size distribution was evaluated by running the sample on the FemtoPulse system.

RNA was extracted from abdomen tissue of iyNetDila1 in the Tree of Life Laboratory at the WSI using the RNA Extraction: Automated MagMax™ *mir*Vana protocol ( do Amaral *et al.*, 2023). The RNA concentration was assessed using a Nanodrop spectrophotometer and a Qubit Fluorometer using the Qubit RNA Broad-Range Assay kit. Analysis of the integrity of the RNA was done using the Agilent RNA 6000 Pico Kit and Eukaryotic Total RNA assay.

Protocols developed by the WSI Tree of Life laboratory are publicly available on protocols.io ( Denton *et al.*, 2023).

### Sequencing

Pacific Biosciences HiFi circular consensus DNA sequencing libraries were constructed according to the manufacturers’ instructions. DNA sequencing was performed by the Scientific Operations core at the WSI on Pacific Biosciences SEQUEL II (HiFi) instrument. Hi-C data were also generated from head tissue of iyNetDila1 using the Arima v2 kit. The Hi-C sequencing was performed using paired-end sequencing with a read length of 150 bp on the Illumina NovaSeq 6000 instrument.

### Genome assembly and curation

Assembly was carried out with Hifiasm ( Cheng *et al.*, 2021) and haplotypic duplication was identified and removed with purge_dups ( Guan *et al.*, 2020). The assembly was then scaffolded with Hi-C data ( Rao *et al.*, 2014) using YaHS ( Zhou *et al.*, 2023). The assembly was checked for contamination and corrected as described previously ( Howe *et al.*, 2021). Manual curation was performed using HiGlass ( Kerpedjiev *et al.*, 2018) and PretextView ( Harry, 2022). The mitochondrial genome was assembled using MitoHiFi ( Uliano-Silva *et al.*, 2023), which runs MitoFinder ( Allio *et al.*, 2020) or MITOS ( Bernt *et al.*, 2013) and uses these annotations to select the final mitochondrial contig and to ensure the general quality of the sequence. Table 3 contains a list of relevant software tool versions and sources.

**Table 3.** Software tools and versions used.

Software tool	Version	Source
BlobToolKit	3.5.2	Challis *et al.*, 2020
Hifiasm	0.16.1-r375	Cheng *et al.*, 2021
HiGlass	1.11.6	Kerpedjiev *et al.*, 2018
MitoHiFi	2	Uliano-Silva *et al.*, 2023
PretextView	0.2	Harry, 2022
purge_dups	1.2.3	Guan *et al.*, 2020
YaHS	yahs-1.1.91eebc2	Zhou *et al.*, 2023

### Evaluation of final assembly

A Hi-C map for the final assembly was produced using bwa-mem2 ( Vasimuddin *et al.*, 2019) in the Cooler file format ( Abdennur & Mirny, 2020). To assess the assembly metrics, the *k*-mer completeness and QV consensus quality values were calculated in Merqury ( Rhie *et al.*, 2020). This work was done using Nextflow ( Di Tommaso *et al.*, 2017) DSL2 pipelines “sanger-tol/readmapping” ( Surana *et al.*, 2023a) and “sanger-tol/genomenote” ( Surana *et al.*, 2023b). The genome was analysed within the BlobToolKit environment ( Challis *et al.*, 2020) and BUSCO scores ( Manni *et al.*, 2021; Simão *et al.*, 2015) were calculated.

### Wellcome Sanger Institute – Legal and Governance

The materials that have contributed to this genome note have been supplied by a Darwin Tree of Life Partner. The submission of materials by a Darwin Tree of Life Partner is subject to the **‘Darwin Tree of Life Project Sampling Code of Practice’**, which can be found in full on the Darwin Tree of Life website here. By agreeing with and signing up to the Sampling Code of Practice, the Darwin Tree of Life Partner agrees they will meet the legal and ethical requirements and standards set out within this document in respect of all samples acquired for, and supplied to, the Darwin Tree of Life Project.

Further, the Wellcome Sanger Institute employs a process whereby due diligence is carried out proportionate to the nature of the materials themselves, and the circumstances under which they have been/are to be collected and provided for use. The purpose of this is to address and mitigate any potential legal and/or ethical implications of receipt and use of the materials as part of the research project, and to ensure that in doing so we align with best practice wherever possible. The overarching areas of consideration are:

•      Ethical review of provenance and sourcing of the material

•      Legality of collection, transfer and use (national and international)

Each transfer of samples is further undertaken according to a Research Collaboration Agreement or Material Transfer Agreement entered into by the Darwin Tree of Life Partner, Genome Research Limited (operating as the Wellcome Sanger Institute), and in some circumstances other Darwin Tree of Life collaborators.

## Data Availability

European Nucleotide Archive: *Netelia fuscicornis*. Accession number PRJEB54053; https://identifiers.org/ena.embl/PRJEB54053. ( Wellcome Sanger Institute, 2022) The genome sequence is released openly for reuse. The *Netelia fuscicornis* genome sequencing initiative is part of the Darwin Tree of Life (DToL) project. All raw sequence data and the assembly have been deposited in INSDC databases. The genome will be annotated using available RNA-Seq data and presented through the Ensembl pipeline at the European Bioinformatics Institute. Raw data and assembly accession identifiers are reported in Table 1.

## Author information

Members of the Natural History Museum Genome Acquisition Lab are listed here: https://doi.org/10.5281/zenodo.7139035.

Members of the Darwin Tree of Life Barcoding collective are listed here: https://doi.org/10.5281/zenodo.4893703.

Members of the Wellcome Sanger Institute Tree of Life Management, Samples and Laboratory team are listed here: https://doi.org/10.5281/zenodo.10066175.

Members of Wellcome Sanger Institute Scientific Operations: Sequencing Operations are listed here: https://doi.org/10.5281/zenodo.10043364.

Members of the Wellcome Sanger Institute Tree of Life Core Informatics team are listed here: https://doi.org/10.5281/zenodo.10066637.

Members of the Tree of Life Core Informatics collective are listed here: https://doi.org/10.5281/zenodo.5013541.

Members of the Darwin Tree of Life Consortium are listed here: https://doi.org/10.5281/zenodo.4783558.
