# Supplementary material for: The genome sequence of an ichneumonid wasp, Netelia fuscicornis (Holmgren, 1860)
Source: Wellcome Open Res. 2024 Jul 10;9:363. [Version 1] doi: 10.12688/wellcomeopenres.22578.1 (PMC11484535; doi:10.12688/wellcomeopenres.22578.1)
Supplement: Peer review history

# Reviewer report: Reviewer response for version 1

**DOI:** 10.21956/wellcomeopenres.24876.r103396

**Published:** 2024-10-21

**Authors:** James Whitfield

**Affiliations:** 1 University of Illinois at Urbana-Champaign, Champaign, USA

This manuscript describes a newly obtained sequence and genome assembly for a common and widespread nocturnally active ichneumonid wasp species. The methodology used appears to be relatively state of the art and the resulting genome assembly is of reasonably high quality.  The data are publicly available, and plans are detailed for future annotation of the genome.

 Relative to several other genome reports I have read in this series, this one provides a rather nice introduction to the species and to why this genome will be useful in comparative studies.  The wasp species is unusual in being a koinobiont ectoparasitoid (most koinobionts being endoparasitic), so the traits *Netelia *species have might prove especially interesting.

Are sufficient details of methods and materials provided to allow replication by others?

Yes

Is the rationale for creating the dataset(s) clearly described?

Yes

Are the datasets clearly presented in a useable and accessible format?

Yes

Are the protocols appropriate and is the work technically sound?

Yes

Reviewer Expertise:

parasitoid wasp ecology and systematics, and evolution of symbiotic viruses

I confirm that I have read this submission and believe that I have an appropriate level of expertise to confirm that it is of an acceptable scientific standard.

# Reviewer report: Reviewer response for version 1

**DOI:** 10.21956/wellcomeopenres.24876.r103400

**Published:** 2024-10-16

**Authors:** Jesper Bechsgaard

**Affiliations:** 1 Aarhus University, Aarhus, Denmark

Price and Broad describe the sequencing and assembly of a high quality reference genome of the ichneumonid was, *Netelia fuscicornis*. The reference genome was constructed using high quality long read sequencing (PacBio HiFi) and chromosome confirmation Hi-C data (Arima), which were analyzed using Hifiams, purge_dups and YaHS. The assembly generally meets the current quality benchmarking thresholds.

Are sufficient details of methods and materials provided to allow replication by others?

Yes

Is the rationale for creating the dataset(s) clearly described?

Partly

Are the datasets clearly presented in a useable and accessible format?

Yes

Are the protocols appropriate and is the work technically sound?

Yes

Reviewer Expertise:

Population genomics

I confirm that I have read this submission and believe that I have an appropriate level of expertise to confirm that it is of an acceptable scientific standard.

# Reviewer report: Reviewer response for version 1

**DOI:** 10.21956/wellcomeopenres.24876.r92877

**Published:** 2024-09-06

**Authors:** Sara E Miller

**Affiliations:** 1 Cornell University, University of Missouri St. Louis, St Louis, Missouri, USA

The authors present a new high quality nuclear and mitochondrial genome assembly for the ichneumonid wasp *Netelia fuscicornis *(Hymenoptera: Ichneumonidae). This widespread species is an ectoparasitoid of Lepidoptera larvae (caterpillars). 

 The genome was constructed for single female using Pacific Biosciences HiFi circular consensus DNA libraries, sequenced on the Biosciences SEQUEL II, and scaffolded with Hi-C data. Raw data and assembly methods are available. The final assembly is 324.7 Mb in length with a scaffold N50 of 47.3 Mb. The genome assembly has 95.2% coverage of BUSCO genes, however there is no annotation currently available for this assembly. 

 Ichneumonid wasps are one of the most specious groups of Hymenoptera, but are underrepresented in current Hymenoptera genome assemblies. This new, high-quality assembly of *N. fuscicornis,* increases our understanding of this understudied group.

Are sufficient details of methods and materials provided to allow replication by others?

Yes

Is the rationale for creating the dataset(s) clearly described?

Yes

Are the datasets clearly presented in a useable and accessible format?

Yes

Are the protocols appropriate and is the work technically sound?

Yes

Reviewer Expertise:

Social insect genetics, and genomics, evolutionary ecology.

I confirm that I have read this submission and believe that I have an appropriate level of expertise to confirm that it is of an acceptable scientific standard.
